# The activity of CouR, a MarR family transcriptional regulator, is modulated through a novel molecular mechanism

**DOI:** 10.1093/nar/gkv955

**Published:** 2015-09-22

**Authors:** Hiroshi Otani, Peter J. Stogios, Xiaohui Xu, Boguslaw Nocek, Shu-Nan Li, Alexei Savchenko, Lindsay D. Eltis

**Affiliations:** 1 Department of Microbiology and Immunology, Life Sciences Institute, The University of British Columbia, Vancouver, British Columbia V6T 1Z3, Canada; 2 Department of Chemical Engineering and Applied Chemistry, University of Toronto, Toronto, Ontario M5S 3E5, Canada; 3 Structural Biology Center, Biosciences Division, Argonne National Laboratory and the Midwest Center for Structural Genomics, Lemont, IL 60439, USA

## Abstract

CouR, a MarR-type transcriptional repressor, regulates the *cou* genes, encoding *p*-hydroxycinnamate catabolism in the soil bacterium *Rhodococcus jostii* RHA1. The CouR dimer bound two molecules of the catabolite *p*-coumaroyl–CoA (*K*_d_ = 11 ± 1 μM). The presence of *p*-coumaroyl–CoA, but neither *p*-coumarate nor CoASH, abrogated CouR's binding to its operator DNA *in vitro*. The crystal structures of ligand-free CouR and its *p*-coumaroyl–CoA-bound form showed no significant conformational differences, in contrast to other MarR regulators. The CouR–*p*-coumaroyl–CoA structure revealed two ligand molecules bound to the CouR dimer with their phenolic moieties occupying equivalent hydrophobic pockets in each protomer and their CoA moieties adopting non-equivalent positions to mask the regulator's predicted DNA-binding surface. More specifically, the CoA phosphates formed salt bridges with predicted DNA-binding residues Arg36 and Arg38, changing the overall charge of the DNA-binding surface. The substitution of either arginine with alanine completely abrogated the ability of CouR to bind DNA. By contrast, the R36A/R38A double variant retained a relatively high affinity for *p*-coumaroyl–CoA (*K*_d_ = 89 ± 6 μM). Together, our data point to a novel mechanism of action in which the ligand abrogates the repressor's ability to bind DNA by steric occlusion of key DNA-binding residues and charge repulsion of the DNA backbone.

## INTRODUCTION

The MarR family of transcriptional regulators, named after ‘multiple antibiotic resistance regulators’, comprises over 12 000 members widely distributed in bacteria and archaea and controls the expression of genes involved in a myriad of cellular processes, from metabolism to virulence (1). For example, PcaV regulates the protocatechuate catabolic genes in *Streptomyces coelicolor* A3(2) (2), MexR regulates multidrug efflux genes in *Pseudomonas aeruginosa* to confer antibiotic resistance (3,4) and OhrR regulates organic hydroxide resistance genes in *Bacillus subtilis* and *Xanthamonas campestris* (5–8). Finally, in pathogenic bacteria, such as *Salmonella enterica* serovar Typhimurium and *Dickeya dadantii*, SlyA confers resistance to antibiotics, antimicrobial peptides and oxidative agents, allowing proliferation in macrophages (9–11).

MarR family regulators function as homodimers (1,12,13). The protomer has a mainly α-helical structure with a triangular topology (14) and the DNA-binding motif is a winged helix-turn helix (wHTH). The regulator binds an inverted repeat nucleic acid sequence or ‘box’, with each of the two wHTH motifs of the dimer binding one of the repeats. Most MarR-family members characterised to date repress transcription, although some are activators (15–17).

The DNA-binding activity of MarR-family members is typically ligand-responsive, with the ligand often being an antibiotic or a phenolic compound. Alternatively, DNA-binding can be modulated by the oxidation of cysteine residues in the regulator. Regardless of the nature of the event that modulates the MarR repressor's DNA-binding activity, the studies to date have established a paradigm whereby the regulator's affinity for its box is relieved by a change in the relative orientation of the wHTH motifs. More specifically, the wHTH motifs have been observed to rotate upwards towards the dimerization interface so that the two motifs, and especially their DNA recognition helices, are no longer able to productively bind the DNA. In PcaV, for example, the binding of protocatechuate induces a 15° rotation of the wHTH towards the dimerization interface (2). In MexR, a similar conformational change is induced by ArmR, a 53-residue antirepressor (18,19). In OhrR, the oxidation of key cysteine residues by organic hydroperoxides results in a 28° rotation of the wHTH motifs (5–8). In the prototypical MarR of *Escherichia coli*, it was recently established that the regulator senses intracellular copper(II) which oxidizes a cysteine residue to generate disulphide bonds between MarR dimers (20). The resulting dimer of dimers dissociates from the MarR box. Finally, ligand-induced structural change can also increase the MarR family regulator's affinity for DNA as exemplified by AdcR, the adhesin competence regulator of *Streptococcus pneumoniae*. In this case, Zn(II)-binding induces a global structural change in AdcR that increases the regulator's affinity for its operator sequence (21).

**Figure 1.**
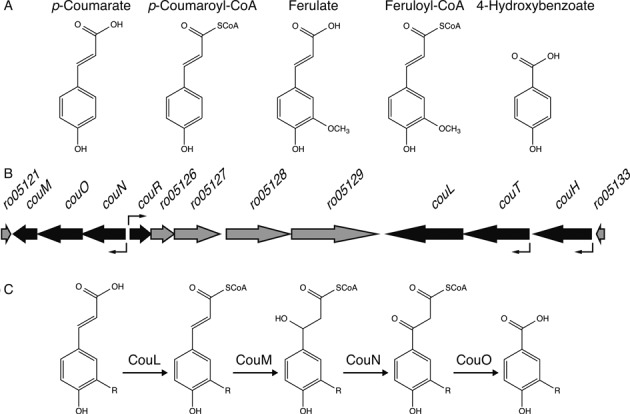
*p*-Hydroxycinnamate catabolism in *Rhodococcus jostii* RHA1. (**A**) Structures of aromatic compounds used in this study. (**B**) The *cou* gene cluster. Black arrows indicate the genes involved in the β-oxidative deacetylation of *p*-hydroxycinnamate. These genes are upregulated in the presence of *p*-hydroxycinnamate. The bent arrows indicate the transcriptional start sites. (**C**) The β-oxidative deacetylation pathway of *p*-hydroxycinnamate.

The soil Actinobacterium *Rhodococcus jostii* RHA1 (RHA1) is able to grow on a wide variety of aromatic compounds, including *p*-hydroxycinnamates (22), such as ferulate and *p*-coumarate (Figure 1A). The catabolism of such compounds is of interest due to their commercial value as antioxidants and precursors for antimicrobial compounds (23,24). In RHA1, the catabolism of *p*-hydroxycinnamates is specified by the *cou* genes (Figure 1B). As in other *p*-hydroxycinnamate-degrading bacteria, this catabolism is initiated by the CoA-thioesterification of the substrate followed by the β-oxidative of the side chain. In RHA1, the last step of this deacetylation is unusual in that it is catalyzed by a member of the amidohydrolase superfamily, CouO and yields an aromatic acid (Figure 1C). These *p*-hydroxybenzoates are converted to protocatechuate and degraded via the β-ketoadipate pathway (25,26).

The *cou* gene cluster includes *couR*, predicted to encode a MarR-family transcriptional regulator. CouR shares ∼25% amino acid sequence identity with FerC and CouR_Rpa_ which regulate *p*-hydroxycinnamate catabolism in *Sphingobium* sp. SYK-6 and *Rhodopseudomonas palustris*, respectively (27,28). In these repressors, DNA-binding is abolished by *p*-hydroxycinnamoyl–CoA, the first metabolite of the pathway, enabling transcription of the catabolic genes. However, the molecular mechanism by which the CoA-thioester relieves DNA-binding in these regulators has yet to be elucidated.

Herein, we used molecular genetic, biochemical, biophysical and structural analyses to gain molecular knowledge of the function of the CouR transcriptional regulator from RHA1. The CouR regulon was defined by mapping CouR-binding sites to the *cou* promoters and demonstrating that CouR acts as a repressor. We used gel shift assays and isothermal titration calorimetry (ITC) to establish that *p*-hydroxycinnamoyl–CoAs relieve the DNA-binding activity of CouR. Structural characterization followed by mutagenesis were then used to investigate the mechanism of how the binding of *p*-hydroxycinnamoyl–CoA to CouR attenuates the latter's DNA-binding activity. Our studies establish a previously unreported mechanism by which ligand-binding abrogates DNA-binding in a transcriptional repressor and provide novel insights into the molecular basis of ligand-mediated attenuation of DNA-binding by MarR-family proteins.

## MATERIALS AND METHODS

### Bacterial strains, plasmids and growth conditions

*Rhodococcus* strains were routinely cultivated at 30°C in 50 ml M9 mineral media supplemented with trace metals (M9 + G (29)) and 2 mM of organic growth substrate. Cultures were inoculated with 5 μl of a cell suspension prepared by growing the cells on 10 mM glucose M9 + G for 2 days, harvesting them by centrifugation and suspending them in the same volume of M9 + G. *E. coli* was grown as described by Green and Sambrook (30). Media were supplemented with ampicillin (50 mg l^−1^), kanamycin (50 mg l^−1^), neomycin (10 mg l^−1^) and apramycin (50 mg l^−1^) as appropriate.

### DNA manipulation and generation of mutants

DNA was propagated, amplified, purified, digested and ligated using standard protocols (30). Oligonucleotides used in this study are listed in Supplementary Table S1. Strains and plasmids used and generated in this study are listed in Supplementary Table S2.

To clone *couR*, the gene was amplified using primers *couR*-F and *couR*-R. The amplicon was cloned into pColdI using NdeI and BamHI to yield pCo*couR*. For crystallographic studies, *couR* was subcloned from pCo*couR* into p15Tv-LIC (31), which codes for a fusion protein containing an N-terminal His_6_ tag, a TEV protease cleavage site, followed by CouR. The pCo*couR*36A, pCo*couR*38A and pCo*couR*36A38A constructs were generated using polymerase chain reaction (PCR)-based directed mutagenesis and pCo*couR* (described below) as the template together with one of three pairs of oligonucleotides: *couR*36A_S and *couR*36A_A; *couR*38A_S and *couR*38A_A; and *couR*36A38A_S and *couR*36A38A_A. The amplified DNA was treated with *Dpn*I (New England BioLabs^®^) and introduced into *E. coli* DH5α.

The Δ*couR* mutant strain was constructed using homologous recombination (32). The upstream and downstream regions of *couR* were amplified from RHA1 genomic DNA using the primer pairs *couR*-FF plus *couR*-FR and *couR*-RF plus *couR*-RR, respectively. The amplicons were combined using splicing by overlap extension-PCR (SOE-PCR). The combined fragment was cloned into pK18*mobsacB* using EcoRI and HindIII to yield pK18Δ*couR*. RHA1 cells were transformed with pK18Δ*couR* by electroporation (33). Neomycin-resistant colonies resulting from a single cross-over were isolated and replica-plated on LB supplemented with 10% (w/v) sucrose. Sucrose-resistant colonies resulting from a second cross-over were isolated and deletion of *couR* was confirmed using PCR (data not shown).

Complementation was performed using pSET152, an integration vector (34). The *couR* gene was amplified from RHA1 genomic DNA using the primers *couR*_c_F plus *couR*_c_R. The amplicon was cloned into pSET152 using EcoRI and XbaI. The pSET*couR*36A38A construct was generated using PCR-based directed mutagenesis, pSET*couR* as the template, and the oligonucleotides *couR*36A38A_S and *couR*36A38A_A. The amplified DNA was treated with DpnI (New England BioLabs^®^) and introduced into *E. coli* DH5α. The resulting plasmids were integrated into the genomes of the Δ*couR* strains as appropriate.

### Purification of CouR and its variants

*Escherichia coli* BL21(DE3) cells freshly transformed with either pCo*couR*, pCo*couR*36A, pCo*couR*38A or pCo*couR*36A38A were grown in 50 ml LB at 30°C overnight. One litre of fresh LB was inoculated with 10 ml of the overnight culture and incubated at 30°C. After 2 h, isopropyl β-D-1-thiogalactopyranoside (IPTG) was added to a final concentration of 0.2 mM and incubation was continued for 24 h at 16°C. Cells were harvested by centrifugation, suspended in 20 mM 4-(2-hydroxyethyl)-1-piperazineethanesulfonic acid (HEPES), pH 7.5, and 300 mM sodium chloride and then lyzed at 4°C using an Emulsi Flex-C5 homogenizer (Avestin Inc.). The His-tagged protein was purified from the supernatant using a column with Ni-NTA Agarose (QIAGEN) according to the manufacturer's instructions. The affinity tag was removed by incubating ∼5 mg ml^−1^ CouR with 50 μg ml^−1^ Factor Xa (New England BioLabs^®^) at 15°C overnight in 50 mM Tris, pH 8 containing 0.1 M magnesium chloride and 1 mM calcium chloride. The digested CouR was further purified using anion exchange and size exclusion chromatographies. The protein was loaded onto a Mono Q GL (GE Healthcare) equilibrated with 20 mM HEPES, pH 7.0 and CouR was eluted using a linear gradient from 0 to 0.5 M sodium chloride in 50 ml. Fractions containing CouR were pooled, dialyzed against 20 mM HEPES, pH 7.0 and 50 mM potassium chloride, concentrated to ∼1 ml and loaded onto a Superdex 75 10/300 equilibrated with 20 mM HEPES, pH 7.0 containing 50 mM potassium chloride. Fractions containing CouR were pooled and concentrated to ∼50 mg ml^−1^ and stored at −80°C until use. CouR-containing fractions were evaluated using sodium dodecyl sulphate-polyacrylamide gel electrophoresis. The concentration of CouR in purified preparations was determined using a molar absorptivity (ϵ_280_) of 2.68 mM^−1^ cm^−1^ in 6 M guanidine hydrochloride (35). The selenomethionine-substituted protein was produced using the pTv-LIC construct transformed into *E. coli* BL21(DE3) codon plus cells. Cells were grown on M9 medium using the high yield procedure according to the manufacturer's instructions (Shanghai Medicilon).

### Electrophoretic mobility shift assay (EMSA)

Electrophoretic mobility shift assays (EMSAs) were performed with a DIG gel shift kit 2nd generation (Roche). Four probes were prepared using the following pairs of oligonucleotides: *couR*p_*couN*p, *couR*p_*couN*p_S and *couR*p_*couN*p_A; *couR*p_*couN*p_m, *couR*p_*couN*p_m_S and *couR*p_*couN*p_m_A; *couT*p, *couT*p_S and *couT*p_A; and *couH*p, *couH*p_S and *couH*p_A. Each pair was annealed by heating at 95°C for 5 min and slowly cooling to 25°C. The 3′ ends of the fragments were then labelled with digoxigenin (DIG)-11-ddUTP. DNA binding assays were performed by incubating 25 to 200 nM CouR, 1 nM DIG-labelled probe and 100 μg ml^−1^ poly[d(I-C)] in 10 μl 20 mM HEPES, 10 mM (NH_4_)_2_SO_4_, 1 mM dithiothreitol, 0.2% (w/v) Tween 20, 30 mM KCl and 1 mM ethylenediaminetetraacetic acid (EDTA), pH 7.6 for 20 min at 25°C. The samples were resolved using electrophoresis and the DNA was electroblotted onto a Hybond-N+ nylon membrane (GE Healthcare) in 0.5× Tris-borate-EDTA buffer (44.5 mM Tris/HCl, 44.5 mM boric acid and 1 mM EDTA, pH 8.0). The DIG-labelled probes were detected by chemiluminescence according to the manufacturer's instructions. Assays were also performed in the presence of 500 μM ligand or 1 μM unlabelled DNA. In the assays, a mixture containing 200 nM CouR and 1 nM DIG-labelled *couR*p*_couN*p probe was supplemented with either CoASH, *p*-coumarate, *p*-coumarate + CoASH, *p*-coumaroyl–CoA, ferulate, ferulate + CoASH, feruloyl–CoA, acetyl–CoA or unlabelled DNA probe.

### Isothermal titration calorimetry (ITC)

Experiments were conducted using a MicroCal iTC_200_ (GE Healthcare) operated at 25°C. Ligands and proteins were loaded into the sample cell and injection syringe, respectively and were in 20 mM HEPES, pH 7.0 and 50 mM potassium chloride. Titrations were performed using 1 mM CouR, 1.5 mM CouR R36A, 1.5 mM CouR R38A and 3 mM CouR R36A/R38A with 0.1 mM, 0.15 mM, 0.15 mM or 0.3 mM *p*-coumaroyl–CoA, respectively. Additional titrations were performed using 1.5 mM CouR and either 0.15 mM *p*-coumarate or 0.15 mM CoASH. Each ITC run comprised an initial injection of 0.4 μl followed by 19 × 2 μl injections of CouR into the sample cell. Experiments were performed in triplicate. Data were analysed using Origin 7.0 software by fitting a titration curve to the corrected data using a single-site interaction model (MicroCal).

### CouR crystallization

Crystals of ligand-free CouR were obtained using the hanging drop vapour diffusion method by mixing 2 μl of 25 mg ml^−1^ selenomethionine-derivatized protein with 2 μl of reservoir solution (0.2 M magnesium acetate, 4% (w/v) glycerol and 20% (w/v) PEG3350) at room temperature. For crystallization of the CouR-*p*-hydroxycinnamol–CoA complex, 80 μl of native protein at 21 mg ml^−1^ were preincubated with 20 μl of 25 mM *p*-hydroxycinnamoyl–CoA. The crystals of the binary complex were obtained using the hanging drop vapour diffusion method by mixing 2 μl of the protein–ligand mix with 2 μl of reservoir solution (0.2 M magnesium acetate, 0.1 M sodium cacodylate pH 6.5, 4% (w/v) 2-methyl-2,4-pentanediol and 26% (w/v) PEG 8K) at room temperature. Crystals were cryoprotected with paratone oil and flash frozen in liquid nitrogen. X-ray diffraction data for CouR was collected at 100 K at the Advanced Photon Source Structural Biology Center beamline 19-ID at wavelength 0.9794 Å using an ADSC Quantum Q315r detector. X-ray diffraction data for the CouR–*p*-hydroxycinnamoyl–CoA complex were collected at 100 K using a Rigaku HomeLab system featuring Micromax-007 HF rotating copper anode fitted with a Rigaku R-AXIS IV++ image plate detector. Diffraction data were processed and reduced using the HKL-3000 software package (36). The CouR structure was solved by the single anomalous dispersion (SAD) method using the Shelx software package (37) and mlphare from the CCP4 software package (38). The structure of the CouR–ligand complex was solved by molecular replacement (MR) using the CouR structure as the search query and Phenix.phaser (39). Structures were refined using Phenix and Coot (40). The presence of additional non-protein electron density corresponding to two molecules of *p*-hydroxycinnamoyl–CoA in the AU of the binary complex was verified by first deleting both ligand molecules plus all atoms within 5 Å of them, followed by simulated annealing (Cartesian) omit maps using Phenix.refine with default parameters. The ligands were built into the resulting residual positive *F*_o_–*F*_c_ density and then occupancy values were refined. The final CouR structure contained four protomers (two dimers) spanning residues 1–142, 4–145, 4–143 and 4–143, with residues 85–95, 89–90, 89–91 and 91–92, in the four respective chains, not modelled due to poor electron density. The final CouR–ligand complex structure contained a dimer and two complete molecules of *p*-coumaroyl–CoA in the AU. Each protomer comprised residues 4–146 except for residues 87–91 of Chain A which were not modelled due to poor electron density. Geometries were verified using Phenix.refine, Coot and the wwPDB Validation server. The PDB accession numbers for the CouR and CouR–*p*-coumaroyl–CoA structures are 3FM5 and 5CYV, respectively.

### Sequence and structural analyses

Amino acid sequence alignments and phylogenetic trees were generated using Clustal Omega (http://www.ebi.ac.uk/Tools/msa/clustalo/). Protein–protein and protein–ligand interfaces were calculating using PDBePISA (41). Electrostatic surfaces of CouR were calculated using Chimera (42). Structure figures were produced with PyMOL and Chimera.

### RNA isolation and real time-quantitative polymerase chain reaction (RT-qPCR)

Total RNA was extracted using TRIzol^®^ Reagent (Invitrogen™) according to the manufacturer's instructions. The RNA was treated with TURBO™ DNase (Invitrogen™) and extracted with phenol–chloroform. cDNAs were synthesized using SuperScript™ III Reverse Transcriptase (Invitrogen™) according to the manufacturer's instructions. Reactions were performed using SYBR^®^ Select Master Mix (Invitrogen™) and the following conditions: 2 min at 50°C and 2 min at 95°C followed by 40 cycles of 15 s at 95°C, 15 s at 60°C and 1 min at 72°C. The internal control was *sigA*. Assays were performed in triplicate using a StepOnePlus™ Real-Time PCR System (Applied Biosystems^®^). The data from each replicate were normalized using the internal standard.

### Size exclusion chromatography-multi angle light scattering (SEC-MALS)

The molecular masses of CouR and CouR–DNA complexes were determined using SEC-MALS. A total of 50 μl of 30 μM CouR in 20 mM HEPES, pH 7.0 and 50 mM potassium chloride was injected into an HPLC 1260 Infinity LC (Agilent Technologies) equipped with a Superdex 200 10/300 column (GE Healthcare). The column was operated at room temperature and a flow rate of 0.2 ml min^−1^. Data were collected with the miniDAWN TREOS multiangle static light scattering device and Optilab T-rEX refractive index detector (Wyatt Technologies). Data were analysed using the ASTRA6 program (Wyatt Technologies).

## RESULTS AND DISCUSSION

### CouR negatively regulates the transcription of *cou* genes

**Figure 2.**
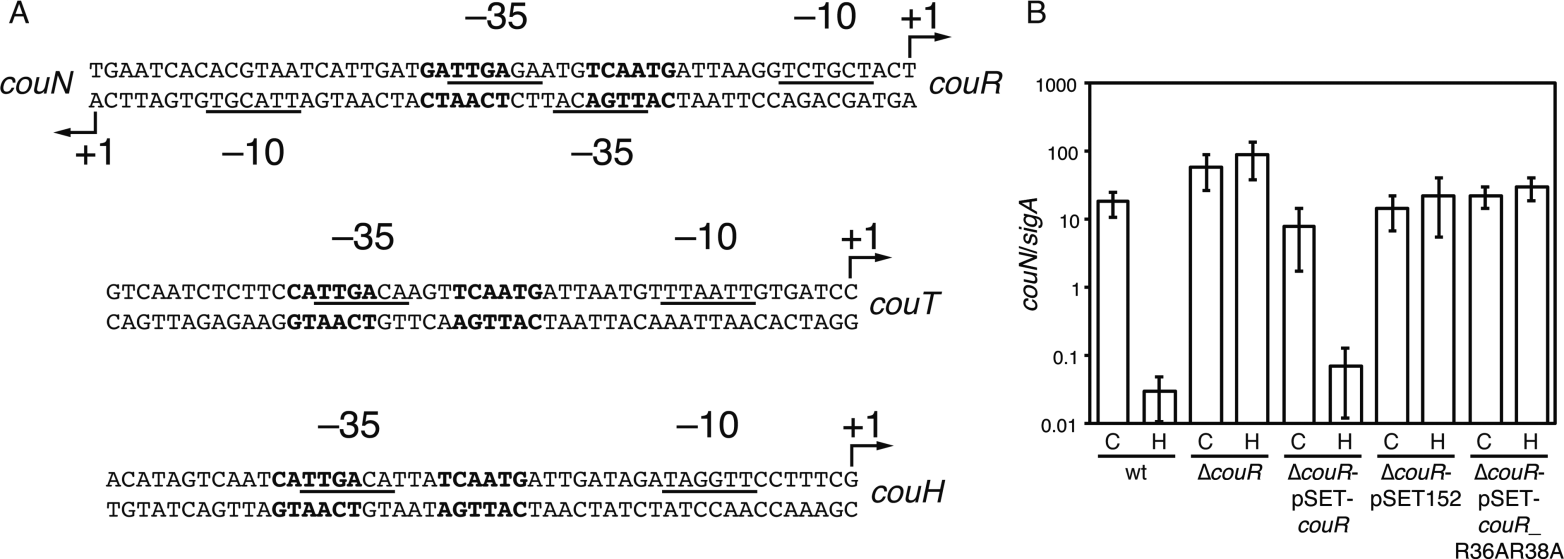
(**A**) The promoter sequences of the CouR regulon. The transcriptional start sites are indicated as +1 with bent arrows. The predicted CouR boxes are indicated in bold. The predicted −10 and −35 boxes are underlined. (**B**) RT-qPCR analysis of *couN*. Cells were grown in the presence of 2 mM *p*-coumarate (C) or 4-hydroxybenzoate (H). The *sigA* gene was used an internal standard. The means and standard deviations from three independent experiments are shown.

Our previous analyses had indicated that the cluster of *cou* genes contains four transcriptional units, with promoters located upstream of *couN, couR, couT* and *couH*, respectively (22). Moreover, transcription from these promoters was activated in the presence of *p*-hydroxycinnamate. To reveal the transcriptional regulatory mechanism of these genes, we first determined their transcriptional start sites using our previous RNA-seq data or 5’ rapid amplification of cDNA ends (5’RACE). The transcriptional start sites of *couN, couR, couT* and *couH* were, respectively, 29, 23, 54 and 47 nt upstream from their initiation codons. Sequence analyses of the four promoters revealed that they all possess canonical −10 and −35 boxes, TtagtT and TTGAcA. Remarkably, they also contain an inverted repeat separated by a 5-bp spacer, cATTGA—–TCAATg, overlapping the predicted −35 box (Figure 2A). Such inverted repeats are characteristic of the nucleotide sequences recognized by MarR family transcriptional regulators (14,27,28,43). The 31 bp stretch between the *couN* and *couR* promoters contains a single inverted repeat that overlaps both predicted −35 boxes, suggesting that a single operator regulates transcription from the divergently transcribed promoters.

The *couR* gene is predicted to encode a MarR family transcriptional regulator, which we hypothesized to regulate the transcription of the *cou* promoters. To test this hypothesis, we deleted *couR* from the RHA1 chromosome and analysed the levels of *couN* transcription using RT-qPCR. In wt RHA1, *couN* transcript levels were 500-fold higher in the presence of *p*-coumarate than 4-hydroxybenzoate (Figure 2B), a growth substrate that is not catabolized by the Cou pathway (22). By contrast, *couN* transcript levels were high in the Δ*couR* strain even in the absence of *p*-coumarate. This phenotype was complemented using pSET*couR*, an actinobacterial integration vector harbouring *couR*, but not the empty vector, pSET152 (Figure 2B). These results demonstrate that CouR negatively regulates *couN* transcription.

**Figure 3.**
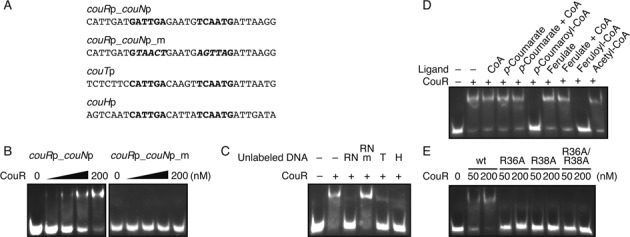
EMSA analyses of CouR. (**A**) DNA sequences used for the EMSA analyses. The predicted CouR boxes are indicated in bold characters. The italicized characters indicate the nucleotide substituted. (**B**) EMSA analysis of the CouR wt to the *couR* and *couN* promoter region. A varying concentration of the CouR wt (0, 25, 50, 100 or 200 nM) was incubated with 1 nM DIG-labelled *couR*p_*couN*p or *couR*p_*couN*p_m probe. (**C**) EMSA analysis of the CouR wt to the *couR*p_*couN*p probe in the presence of the unlabelled *couR*p_*couN*p (RN), *couR*p_*couN*p_m (RNm), *couT*p (T) or *couH*p probe (H). (**D**) EMSA analysis of the CouR wt to the *couR*p_*couN*p probe in the presence of CoASH, *p*-coumarate, ferulate, *p*-coumaroyl–CoA, feruloyl–CoA or acetyl–CoA. (**E**) EMSA analysis of the CouR wt, R36A, R38A and R36A/R38A to the *couR* and *couN* promoter region. A varying concentration of CouR (50 or 200 nM) was incubated with 1 nM DIG-labelled *couR*p-*couN*p probe.

To investigate the involvement of CouR in the regulation of the *cou* genes, we prepared CouR and a predicted CouR box. CouR was produced in *E. coli* with a poly-histidine tag and purified to apparent homogeneity (Supplementary Figure S1A). The purified protein had an N-terminal histidinyl residue not present in wt CouR and, according to SEC-MALS data, was dimeric (Supplementary Figure S2). We also generated a DNA probe, *couR*p_*couN*p, comprising the 31 bp between the *couR* and *couN* promoters, including the inverted repeat predicted to be a CouR box (Figure 3A). In gel shift assays, purified CouR bound to the *couR*p_*couN*p fragment (Figure 3B) but did not bind to a similar fragment, *couR*p_*couN*p_m, in which the 10 nt of the inverted repeat were substituted (Figure 3). These data indicate that the inverted repeat is required for CouR to bind DNA. We therefore annotated this inverted repeat the CouR box.

To further investigate the binding of CouR to the promoters of the CouR regulon, we performed competitive binding studies using a DIG-labelled *couR*p_*couN*p probe and various unlabelled DNA fragments (Figure 3A and C). An excess of unlabelled *couR*p_*couN*p outcompeted DIG-labelled *couR*p_*couN*p. By contrast, an excess of the mutated *couR*p_*couN*p fragment did not sequester CouR. Importantly, unlabelled DNA fragments from the *couT* and *couH* promoters, respectively, that contained the inverted repeat were also able to outcompete the DIG-labelled *couR*p_*couN*p probe (Figure 3C). Overall, these data demonstrate that CouR binds to the promoter regions of the CouR regulon. The data are also consistent with the hypothesis that the binding of CouR to the CouR boxes that overlap the −35 promoter sequences represses the transcription of the four *cou* operons.

### *p*-Hydroxycinnamoyl–CoAs are the ligands of CouR

In general, the ligand-free form of MarR-family repressors bind to cognate nucleotide sequences to repress transcription and this binding is antagonized in the presence of a ligand (1). We therefore sought to identify the CouR ligand. Comparative sequence analysis of CouR against characterized MarR-family proteins indicated that it belongs to a clade that includes FerC and FerR of *Pseudomonas fluorescens* (Supplementary Figure S3), which bind *p*-hydroxycinnamoyl–CoAs, such as *p*-coumaroyl–CoA and feruloyl–CoA (27,28,44). In RHA1, these CoA thioesters are the product of the CouL-catalyzed reaction (Figure 1B). Therefore, we tested the effect of *p*-hydroxycinnamoyl–CoAs on CouR DNA-binding. According to EMSA, 500 μM *p*-coumaroyl–CoA or feruloyl–CoA abolished the DNA-binding ability of CouR (Figure 3D). By contrast, the presence of CoASH, *p*-coumarate, ferulate or acetyl–CoA had no detectable effect on the DNA-binding activity of the transcriptional regulator at the concentrations tested.

**Figure 4.**
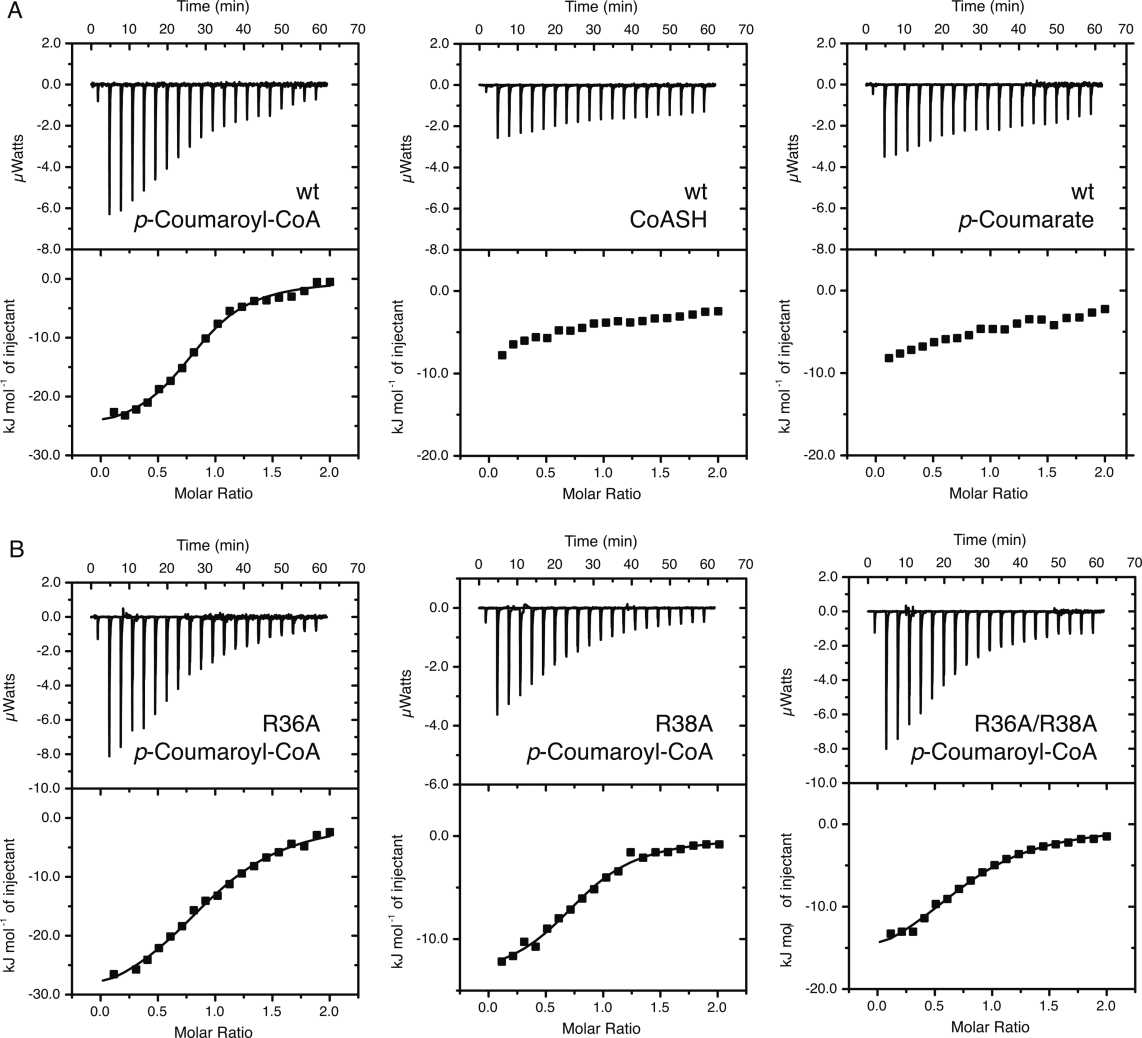
Isothermal calorimetry (ITC) analyses of CouR. (**A**) ITC analysis of wt CouR. About 1 mM CouR was injected into 0.1 mM *p*-coumaroyl–CoA and 1.5 mM CouR was injected into 0.15 mM *p*-coumarate or CoA. (**B**) ITC analysis of the CouR R36A, R38A and R36A/R38A. Experiments were conducted with 1.5 mM CouR R36A with 0.15 mM *p*-coumaroyl–CoA, 1.5 mM CouR R38A with 0.15 mM *p*-coumaroyl–CoA or 3 mM CouR R36A/R38A with 0.3 mM *p*-coumaroyl–CoA. Injection volumes and other details are provided in ‘Materials and Methods’ section.

We then used ITC to further characterize the interaction of CouR with *p*-coumaroyl–CoA (Figure 4A, Table 2). In these experiments, CouR bound *p*-coumaroyl–CoA with a stoichiometry of 1:1 (protomer:ligand) and a dissociation constant of 11 ± 1 μM. No cooperativity was detected in the binding isotherm. Consistent with the EMSA data, CouR did not detectably bind either *p*-coumarate or CoASH. Together, these data demonstrated that the effector of CouR is a *p*-hydroxycinnamoyl–CoA, the product of the CouL-catalyzed reaction.

**Table 1.** Crystallographic data collection and refinement statistics

Structure PDB code	CouR 3FM5	CouR–*p*-coumaroyl–CoA 5CYV
**Data collection**		
Space group	P4_1_2_1_2	C222_1_
Cell dimensions		
*a, b, c*, Å	75.1, 75.1, 213.6	64.1, 134.4, 73.3
Resolution, Å	40.00–1.96	25.00–1.51
R*_merge_^a^*	0.085 (0.603)*^b^*	0.033 (0.470)
*I* / σ(*I*)	23.7 (3.00)	67.0 (3.79)
Completeness,%	97.7 (91.9)	97.8 (83.6)
Redundancy	12.2 (6.8)	6.7 (4.4)

**Refinement**		
Resolution, Å	37.14–2.00	24.79–1.52
No. of reflections: working, test	41566, 2198	48185, 3774
*R*-factor/free *R*-factor^c^	19.6/24.0 (25.6/33.6)	16.1/20.2 (26.7/27.5)
No. of refined atoms		
Protein (chains)	4112 (4)	2171 (2)
Substrate	N/A	118
Magnesium	1	3
Chlorine	1	2
Glycerol+ethylene glycol	26	N/A
Acetate	N/A	4
Water	294	490
*B*-factors, Å^2^		
Protein	55.4	30.2
Substrate	N/A	33.5
Magnesium	53.7	21.9
Chlorine	49.8	34.9
Glycerol + ethylene glycol	66.5	N/A
Acetate	N/A	54.9
Water	50.8	44.5
r.m.s.d.		
Bond lengths, Å	0.004	0.02
Bond angles, °	0.706	1.85

^a^R_merge_ = ∑_hkl_ ∑_j_ | *I*_hkl,j_ - <*I*_hkl_> | / ∑_hkl_ ∑_j_*I*_hkl,j_

^b^Values in parentheses refer to highest resolution shells.

^c^R-factor = ∑_hkl_ | F^o^_hkl_ – F^c^_hkl_ | / ∑_hkl_ F^o^_hkl_.

**Table 2.** Dissociation constants and related thermodynamic parameters of CouR and its variants for *p*-coumaroyl–CoA and related compounds^a,b^

Protein	Compound	N	*K*_d_ (μM)	Δ*H* (kJ/mol)	Δ*S* (J/mol/K)
wt	*p*-coumaroyl–CoA	1.19 (0.01)	11 (1)	−23 (1)	17 (5)
wt	*p*-coumarate	ND	ND	ND	ND
wt	CoASH	ND	ND	ND	ND
R36A	*p*-coumaroyl–CoA	1.04 (0.03)	28 (2)	−33 (1)	−22 (5)
R38A	*p*-coumaroyl–CoA	1.25 (0.09)	25 (5)	−12 (1)	48 (5)
R36AR38A	*p*-coumaroyl–CoA	1.28 (0.02)	89 (6)	−14 (1)	31 (2)

^a^Values represent the mean from three independent experiments. Standard deviation is indicated in parentheses.

^b^ND: No detectable interaction.

### Crystal structure of CouR

**Figure 5.**
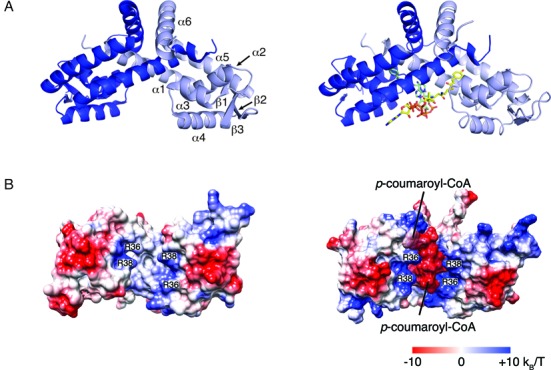
Crystal structures of CouR with and without *p*-coumaroyl–CoA. (**A**) Left panel—one of the dimers of asymmetric unit of the ligand-free structure. Chains B and D are shown in dark and light blue, respectively. Right panel—the CouR–*p*-coumaroyl–CoA dimer. (**B**) Electrostatic surface of the DNA-binding region of CouR without (left) and with (right) *p*-coumaroyl–CoA. Colour-coded from –10 k_B_T e^−1^ to 10 k_B_T e^−1^.

To understand the mechanism by which *p*-hydroxycinnamoyl–CoAs modulate the DNA-binding activity of CouR, the crystal structure of CouR was determined to a resolution of 1.96 Å (Figure 5A; see crystallographic statistics in Table 1). The asymmetric unit (AU) contained two homodimers, annotated as Chains A + C and Chains B + D, respectively (Figure 5A). The structures of the two dimers were very similar, superimposing with an RMSD of 1.04 Å of 241 matching α-carbon atoms. The dimers possessed similar dimerization interfaces, described below, but were rotated ∼5° about this interface with respect to each other (Supplementary Figure S4). More specifically, the α1 helices were rotated ∼5° with respect to each other in the two dimers, indicating some flexibility in the orientation of the two subunits within the dimer. Due to this subtle difference between the two dimers, superposition of individual chains from each dimer yielded a better superposition (RMSD of 0.70 Å over 115 matching α-carbon atoms).

The CouR protomer showed the α1-α2-β1-α3-α4-β2-β3-α5-α6 secondary structure topology typical of MarR-family proteins (14). Accordingly, the α1, α5 and α6 helices of each protomer interdigitated to form a dimerization interface. The interface measured 2124 and 2169 Å^2^, respectively, in each dimer, and comprised predominantly hydrophobic interactions. Neither the dimer-dimer interface observed in the crystal structure nor the crystal packing likely reflects higher order oligomerization states because the largest dimer–dimer interface in the AU was <440 Å^2^. Helices α3 and α4 were the helices of the DNA-binding wHTH motif, while the anti-parallel strands β2 and β3 and their connecting loop constituted the ‘wing’. In the CouR structure, this connecting loop (residues 89–92) appeared disordered and thus was not included in the final model. The CouR dimer contained a deep cleft between the two protomers. The cleft was lined with residues from the α2, α3 and α4 helices and was positively charged (Figure 5B).

**Figure 6.**
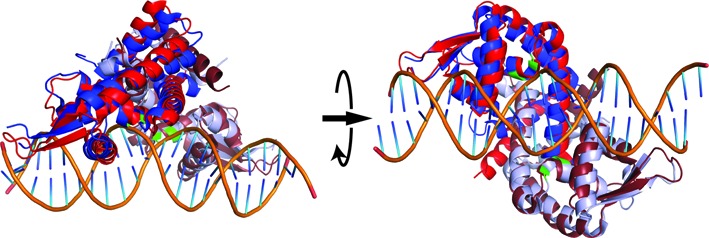
Structural superposition of CouR (blue; Chains B and D) and *Salmonella enterica* SlyA (red) bound to DNA. Regions of the backbone corresponding to Arg36 and Arg38 of CouR are shown in green.

To gain an understanding of CouR interaction with operator DNA, we superimposed the CouR dimer structures onto the dimeric structures of four MarR-family proteins: SlyA (3Q5F), MepR (4LLN), OhrR (1Z9C) and SCO3205 (3ZPL) that were previously structurally characterized in DNA-bound forms (Figure 6 and Supplementary Figure S5; 43,45–47). Of these proteins, CouR was most similar to SlyA: the proteins share 22% amino acid sequence identity and the structures superimposed with RMSD values of 2.91 Å over 240 matching α-carbon atoms and 2.32 Å over 256 matching α-carbon atoms for CouR Chains A + C and Chains B + D, respectively. Accordingly, the binding of CouR to DNA was modelled after the SlyA–DNA complex structure, with the α4 helices and the wings of the DNA-binding motif docked to the major and minor grooves of B-form DNA, respectively. In the SlyA–DNA structure, Thr30 and Thr32 were located in the N-terminus (the α2 helix) and formed hydrogen bonds with the phosphate groups of the DNA (46). These residues corresponded to Arg36 and Arg38 in CouR, suggesting that these residues mediate similar interactions in the presumptive CouR–DNA complex. Notably, the distance between the two Arg36 residues and the two Arg 38 residues in the CouR B + D dimer were 27.5 Å and 23.0 Å, respectively. This is very similar to the distances separating the Thr30 residues (27.8 Å) and the Thr32 residues (22.5 Å) in the DNA-bound SlyA dimer. This indicated that the Arg36 and Arg38 residues of CouR are appropriately positioned to interact with the phosphate backbone of the DNA. This hypothesis is further supported by the high degree of conservation of residues in other MarR transcription repressors that are similarly positioned and interact with the phosphate backbone of the DNA such as Thr39 and Gln42 in OhrR (47), Thr30 and Gln33 in MepR (45), and Thr44 in SCO3205 (43). The high similarity of the CouR structure with the DNA-bound form of SlyA further suggests that (i) the structure of ligand-free CouR is very similar to the DNA-bound conformation of this protein and (ii) the CouR dimer binds to its operator in the same symmetric fashion as SlyA. The latter hypothesis is further supported by the fact that both operators are inverted repeats. More specifically, residues Ser62, Val64 and Arg65 of SlyA interact with base pairs of the SlyA operator. These three are found in the recognition helix of the wHTH motif, and correspond to Asp65, Ser67 and Gln68 in CouR. These residues likely play the same base pair recognition role in CouR as they do in SlyA.

### Ligand binding does not cause a major conformational change in CouR

To determine the molecular basis for recognition of *p*-coumaroyl–CoA by CouR and its potential effects on the conformation of this repressor, we determined a crystal structure of the CouR–*p*-coumaroyl–CoA complex to 1.52 Å resolution (Table 1). The AU of the CouR–*p*-coumaroyl–CoA structure contained a single CouR dimer (Chains A and B) and two molecules of *p*-coumaroyl–CoA (Figure 5A, Supplementary Figure S6A). The stoichiometry of the CouR–ligand structure was consistent with the ITC analysis.

**Figure 7.**
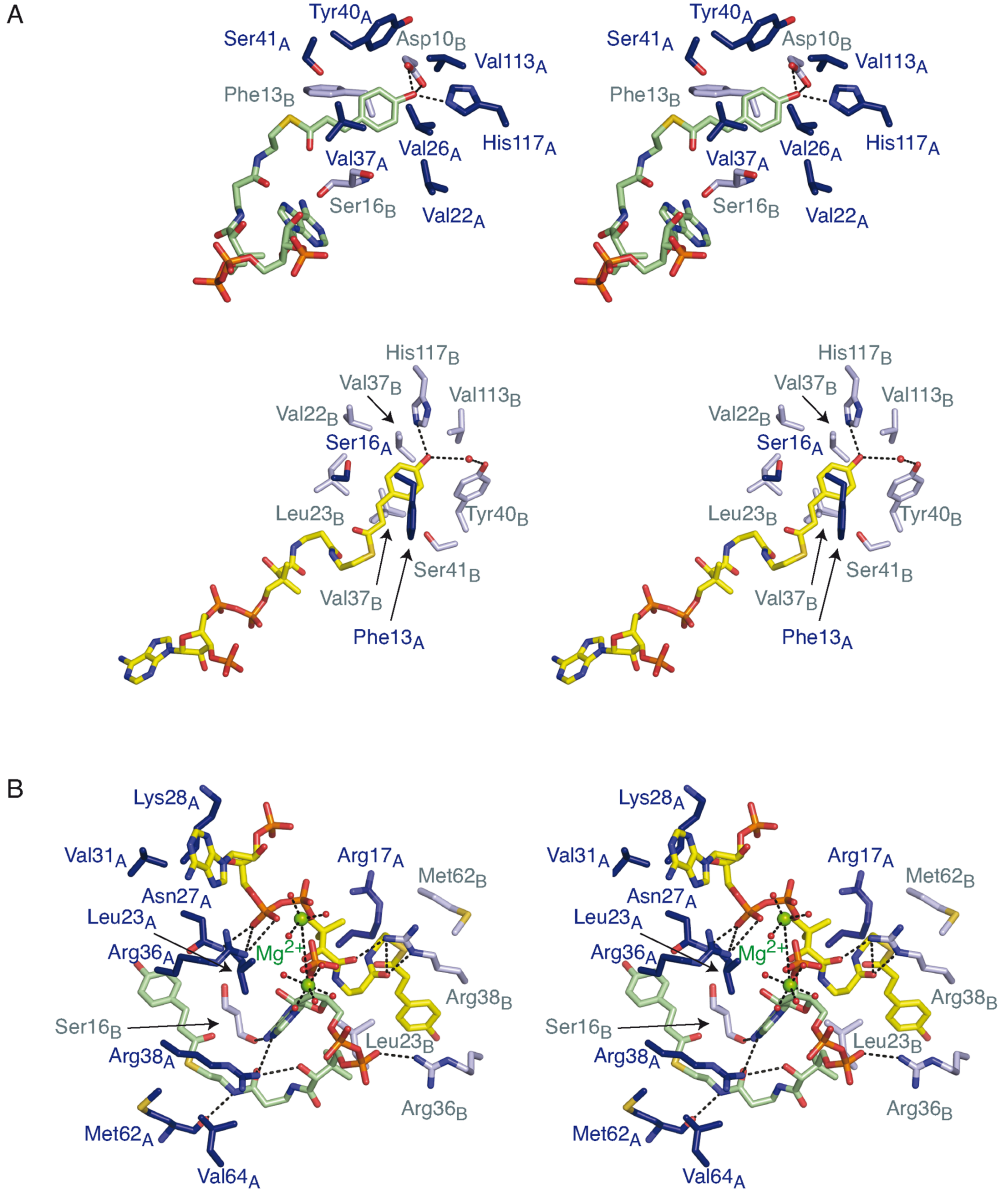
Stereo images of CouR–ligand interactions. (**A**) Binding pockets of the phenolic moieties. Residues and water molecules located within 4 Å of the phenolic moieties of *p*-coumaroyl–CoA are shown in stick representation and red spheres, respectively. (**B**) The CoA-binding site. Residues located within 4 Å from the CoA moieties of *p*-coumaroyl–CoA are shown in stick representation coloured as in A. Water molecules are omitted for clarity except for those coordinating magnesium ions.

The *p*-coumaroyl–CoA ligands bound in the central cavity formed between the two chains of the CouR dimer (Figure 5A). The phenolic moiety of each of the ligands occupied equivalent hydrophobic pockets deep within each CouR protomer (Figure 7A). Each ligand-binding pocket was formed by residues from the α1, α2 and α5 helices of one protomer together with residues from the α1 helix of the other protomer. The pocket-lining residues that contacted the ligand included Asp10, Gly12, Phe13, Ser16 of one protomer and Val22, Leu23, Val26, Val37, Tyr40, Ser41, Val113 and His117 of the other (Figure 7A). Indeed, the phenolic hydroxyl formed hydrogen bonds with Asp10^A^ and His117^B^ of the different protomers (Figure 7A). This binding pocket and the orientation of the phenolic moiety in this pocket were remarkably similar to the interactions between PcaV and its ligand, protocatechuate (2).

In contrast to the buried phenolic moieties, the CoA moieties of the ligands were solvent-exposed on the surface of the groove formed between the two wHTH motifs (Figures 5A and 7B). Strikingly, the two CoA moieties adopted different configurations: one of the ligand molecules was bound in an ‘extended’ configuration along the α1 helix while the other adopted a ‘bent’ conformation with its adenyl group curved back and inserted between the pantothenyl regions of the two ligands (Figure 7B). The *p*-coumaroyl–CoA in ‘extended’ conformation formed hydrogen bonds with Asn27^A^ and Arg36^A^ via its α-phosphate group and with Arg38^B^ via its pantothenyl group (Figure 7B). The bent *p*-coumaroyl–CoA formed hydrogen bonds with Arg38^A^ and Met62^A^ via its pantothenyl group and with Ser16^B^ and Arg36^B^ via the adenyl and phosphate groups, respectively. The bent ligand was less solvent-exposed than the extended one: in the former, 75 atoms and 825 Å were sequestered from the solvent as compared to 73 atoms and 742 Å in the extended ligand. Despite these differences, the six phosphates of the two ligand molecules were nearly collinear, occupying the length of the groove formed between the two protomers of CouR, and were fully solvent exposed. The alignment of the phosphates may be important for abrogating DNA-binding as explained below and could explain the different configurations of the CoA moieties. More specifically, modelling suggests that this alignment cannot be preserved when the two *p*-coumaroyl–CoA molecules are constrained to bind in the same configuration. Indeed, attempts to constrain the two molecules in the same conformation, whether bent or extended, resulted in steric clashes between the ligands as well as between the ligands and the protein. Thus, CouR appears to constrain the ligands to bind in the two different configurations. We also identified additional density that we assigned to two magnesium cations based on their coordination environments and electron density features and because a magnesium salt was present in the crystallization solution; the modelled cations coordinate the phosphates of *p*-coumaroyl–CoA.

Overall, the structural data indicate that the CoA moieties of the two *p*-coumaroyl–CoA ligands bind differently to CouR, but that the phenolic moieties bind in a conserved manner. Consistent with this notion, the phenolic and diphosphate moieties of the ligands have well-ordered electron density and low B-factors (Supplementary Figure S6B). By contrast, the adenosyl moiety of the extended ligand has poorer electron density and elevated B-factors. This apparent stringent recognition of the coumaroyl moieties by CouR appears to constrain the remainder of the ligands such that their diphosphate groups and the 3’-phosphate of the bent ligand are linearly arranged along CouR's central groove and orientated away from the protein.

MarR repressors typically change conformation upon binding their cognate ligands. Accordingly, we searched for such conformational changes by comparing the CouR and CouR–ligand structures (Supplementary Figure S7). Superposition of individual protomers of the CouR dimer (i.e., Chain A or Chain B) with a single protomer of ligand-bound CouR (Chain A) yielded RMSD values of 0.81 and 0.82 Å over 112 and 118 matching α-carbon atoms, respectively, indicating that at the level of a single chain, CouR does not undergo significant conformational change upon binding of *p*-coumaroyl–CoA. In comparing the three dimers, the α1 helices in the CouR–ligand dimer were rotated ∼10° with respect to their positions in either of the ligand-free dimers. However, the partner chain in the ligand-bound CouR (Chain B) occupied a similar position as the equivalent partner chains in the two ligand-free dimers. Reflecting this, 247 Cα atoms of the ligand-bound CouR dimer superposed with ligand-free A + C dimer with an RMSD value of 1.6 Å. Using the B + D dimer, 251 Cα atoms superposed with an RMSD value of 1.5 Å. Most importantly, the relative positions of the α3 and α4 helices of the DNA-binding motifs (i.e., the wHTH) were very similar in the three structures. Thus, the wHTH were rotated ∼4° and ∼6° in the ligand-bound structure as compared to each of the ligand-free structures (Supplementary Figure S7). These conformational differences are modest compared to what has been reported for other MarR family regulators (1). For example, conformational changes in PcaV, MexR and SlyA are associated with 14–21° rotations of the α3 and α4 helices, dramatically lowering the affinity of these regulators for their cognate DNA (Supplementary Figure S7). Significant conformational changes in the case of the PcaV–protocatechuate complex are of particular note given the similar ligand-binding pockets of this protein and CouR. Based on this analysis, we postulated that the affinity of the CouR regulator for its DNA operator sequence is modulated by a mechanism that does not involve ligand-induced conformational changes.

### Arg36 and Arg38 are essential for DNA binding

Having established that binding of *p*-coumaroyl–CoA induces release of CouR from DNA and that this ligand does not induce large-scale conformational changes in the overall structure of the regulator, we hypothesized that competition between the ligand and DNA for the same binding surface on this regulator could mediate this release. Indeed, our structural analyses of the CouR–*p*-coumaroyl–CoA complex (Figure 7) indicated that the CoA moieties overlapped with the predicted DNA binding sites (Figure 6). More specifically, the phosphate moieties of the ligand interacted with Arg36 and Arg38 of CouR. These two residues, located in the wHTH motif, are predicted to form hydrogen bonds with the DNA backbone.

To test the hypothesis that *p*-coumaroyl–CoA physically occludes the DNA binding residues from recognizing the CouR operator box, we substituted Arg36 and Arg38, alone and in combination, with alanine residues and characterized the *p*-coumaroyl–CoA- and DNA-binding capacity of the CouR variants. The CD spectra of the three purified variants were indistinguishable from that of wild-type CouR (Supplementary Figure S1B), suggesting that the substitutions at positions 36 and 38 did not significantly change the protein's overall structure. Substitution of either argininyl residue decreased the affinity of CouR for *p*-coumaroyl–CoA by ∼2.4-fold as compared to the wild type protein (Figure 4B and Table 2). These modest effects were nevertheless cumulative, as the R36A/R38A double variant had an approximately eight-fold lower affinity for the ligand than the wild-type protein. The overall contribution of each Arg residue to ligand binding was ∼2 kJ mol^−1^, which represent ∼7% of the total Gibbs free energy change of the binding reaction. These data support the structural data inasmuch as these residues contribute significantly to ligand-binding but are not the sole binding determinants.

In marked contrast to the ligand-binding data, none of three CouR variants detectably bound the *couR*p_*couN*p DNA fragment in a gel shift assay (Figure 3E). To corroborate this finding, we introduced the allele carrying the R36A/R38A double substitution into the Δ*couR* mutant of RHA1 to assess its function *in vivo*. As shown in Figure 2B, *couN* was expressed to a high level in the variant-complemented mutant during growth on 4-hydroxybenzoate. This result demonstrates that the double variant cannot complement the function of CouR as a repressor. Together, the *in vitro* and *in vivo* experiments establish that Arg36 and Arg38 are major determinants for CouR binding to DNA but are less important for the binding of *p*-coumaroyl–CoA. These data also prompted us to suggest that *p*-hydroxycinnamoyl–CoAs modulate the affinity of CouR for its cognate nucleotide sequence using two mechanisms. First, the CoA moieties sterically and electrostatically occlude the DNA-recognition elements of the CouR dimer from binding DNA; the *p*-coumaroyl–CoA molecule is anchored to CouR through strict recognition of the phenolic moiety. Second, the high local concentration of negative charges of the phosphate groups from the two *p*-coumaroyl–CoA molecules aligned on one face CouR would be expected to repulse the negatively charged backbone of DNA (Figure 5B). The latter of these mechanisms is similar to what has been proposed for CsoR, the copper-sensitive regulator that represents the CsoR family repressors: upon binding Cu(I), negatively charged residues in CsoR's flexible N-terminal tail have been proposed to sequester the basic residues that mediate DNA-binding (48).

The *cou* cluster is conserved in a number of *Actinomycetales* and *Rhizobiales* species, all of which encode a MarR family regulator sharing 19–37% amino acid sequence identity (22). Despite their relatively low sequence identity, these homologues all have one or both of Arg36 or Arg38 (Supplementary Figure S8). In CouR of *Corynebacterium glutamicum* and *Arthrobacter* sp. FB24, the position corresponding to Arg36 is occupied by the chemically-similar lysine residue. Interestingly, neither Arg36 nor Arg38 is conserved in the homologues of *R. palustris* and *Sphingobium* sp. SYK-6 although their DNA-binding is abolished by *p*-hydroxycinnamoyl–CoA (27,28). This suggests that the mechanisms of DNA- and ligand-binding in these regulators are different than in CouR of RHA1. Finally, neither Arg36 nor Arg38 are conserved in PcaV or MobR, regulators which bind protocatechuate and 3-hydroxybenzoate, respectively (2,49).

## CONCLUSION

This study provides important insights into the transcriptional regulatory mechanism of the *cou* genes by CouR as well as the mechanism of the ligand-mediated attenuation of transcriptional repression by MarR family proteins. Although previous biochemical and molecular genetic evidence had established that *p*-hydroxycinnamoyl–CoA could modulate DNA-binding in CouR homologues, its molecular mechanism had yet to be elucidated (27,28). The ITC and structural data establish that the CouR dimer binds two *p*-coumaroyl–CoA molecules in non-equivalent configurations. Importantly, ligand-binding did not lead to a significant conformational change in CouR. This is in marked contrast to what was observed in PcaV despite the protocatechuate-binding pocket of PcaV being highly similar to the *p*-coumaroyl-binding pocket of CouR (2). More significantly, the structural data combined with the functional characterization of the CouR variants strongly indicate that the anionic, bulky CoA moiety of *p*-hydroxycinnamoyl–CoA prevents the binding of DNA by steric occlusion and charge repulsion. This is the first time that this mechanism of modulating DNA affinity has been demonstrated in the MarR family transcriptional regulators. It is unclear how widespread this mechanism occurs in this family: although over 12 000 members have been reported, a relatively limited number of these regulators have been characterized to date. Given their importance in regulating metabolism, pathogenicity and drug resistance in bacteria and archaea, structural and functional characterization of other MarR family members is warranted.

## ACCESSION NUMBERS

PDB IDs: 3FM5 and 5CYV.

## Supplementary Material

SUPPLEMENTARY DATA
