# Supplementary material for: The activity of CouR, a MarR family transcriptional regulator, is modulated through a novel molecular mechanism
Source: Nucleic Acids Res. 2015 Sep 22;44(2):595–607. doi: 10.1093/nar/gkv955 (PMC4737184; doi:10.1093/nar/gkv955)
Supplement: SUPPLEMENTARY DATA [file supp_44_2_595__index.html]

The activity of CouR, a MarR family transcriptional regulator, is modulated through a novel molecular mechanism — SUPPLEMENTARY DATA 

# The activity of CouR, a MarR family transcriptional regulator, is modulated through a novel molecular mechanism

## SUPPLEMENTARY DATA

- SUPPLEMENTARY DATA
