# Supplementary material for: The activity of CouR, a MarR family transcriptional regulator, is modulated through a novel molecular mechanism
Source: Nucleic Acids Res. 2015 Sep 22;44(2):595–607. doi: 10.1093/nar/gkv955 (PMC4737184; doi:10.1093/nar/gkv955)
Supplement: SUPPLEMENTARY DATA [file supp_gkv955_nar-02180-m-2015-File009.pdf]

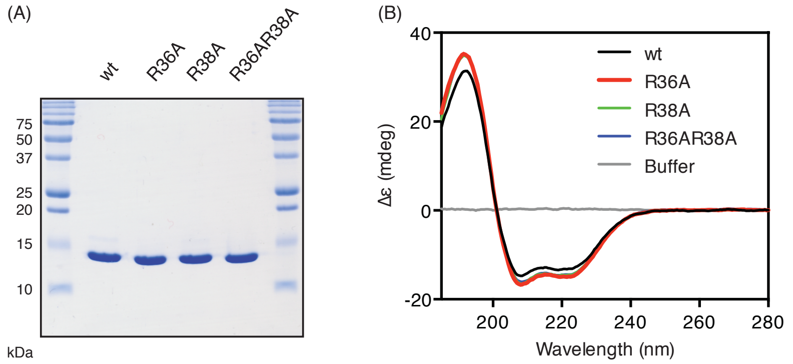

Fig. S1. Purification of CouR. (A) SDS-PAGE of wt CouR and its variants. Lanes contained 2 µg of CouR. (B) Circular dichroic spectra of wt CouR and its variants.

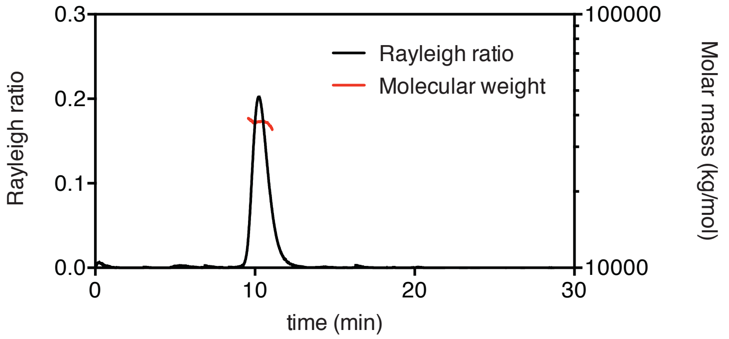

Fig. S2. SEC-MALS of a CouR solution. The Rayleigh ratio is shown as a black line, and the calculated molar mass is shown as a red line. The calculated molar mass was 37.5 kDa, which corresponds to a CouR dimer (31.9 kDa).

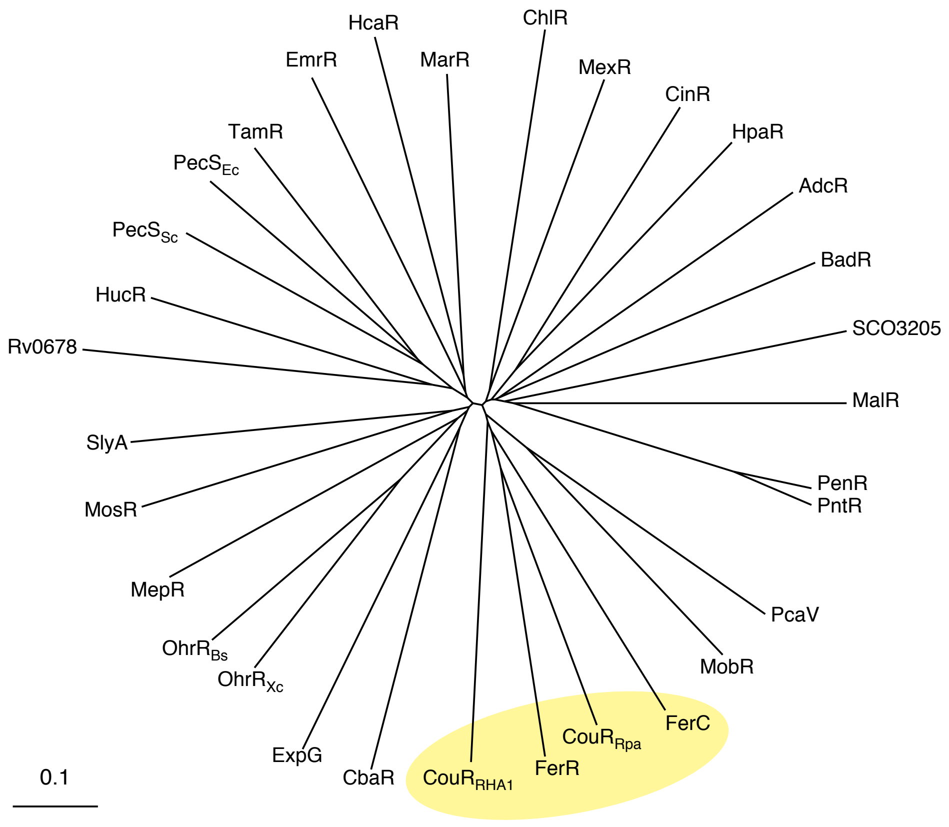

Fig. S3. A guided tree of CouR and characterised MarR-family proteins: CouR of *R. jostii* RHA1, MarR of *Escherichia coli* K-12 (Martin *et al.*, 1996; PDB, 1JGS), HcaR of *Acinetobacter* sp. ADP1 (Parke and Ornston, 2003; PDB, 4RGR), EmrR of *E. coli* K-12 (Lomovskaya *et al.*, 1995), TamR of *Streptomyces coelicolor* A3(2) (Huang and Grove, 2013), PecS of *Erwinia chrysanthemi* 3937 (Rouanet *et al.*, 2004), PecS of *S. coelicolor* A3(2) (Huang *et al.*, 2013), HucR of *Deinococcus radiodurans* R1 (Wilkinson and Grove, 2004; PDB, 2FBK), Rv0678 of *Mycobacterium tuberculosis* H37Rv (Radhakrishnan *et al.*, 2014; PDB, 4NB5), SylA of *Salmonella enterica* Typhimurium LT2 (Dolan *et al.*, 2011; PDB, 3QPT), MosR of *M. tuberculosis* H37Rv (Abomoelak *et al.*, 2009; PDB, 4FX0), MepR of *Staphylococcus aureus* SH1000 (Kumaraswami *et al.*, 2009; PDB, 3ECO), OhrR from *Bacillus subtilis* 168 (Fuangthong *et al.*, 2001; PDB, 1Z91), OhrR of *Xanthomonas campestris* pv. phaseoli (Sukchawalit *et al.*, 2001; PDB, 2PEX), ExpG of *Sinorhizobium meliloti* Rm2011 (Rüberg *et al.*, 1999), CbaR of *Comamonas testosteroni* BR60 (Providenti and Wyndham, 2001), FerR of *Pseudomonas fluorescens* BF13 (Calisti *et al.*, 2008), CouR of *Rhodopseudomonas palustris* CGA009 (Hirakawa *et al.*, 2012), FerC of *Sphingobium* sp. SYK-6 (Kasai *et al.*, 2012), MobR from *C. testosteroni* KH122-3s (Hiromoto *et al.*, 2006), PcaV from *S. coelicolor* A3(2) (Davis *et al.*, 2013; PDB, 4G9Y), PntR from *Streptomyces arenae* TÜ469 (Zhu *et al.*, 2013), PenR from *Streptomyces exfoliatus* UC5319 (Zhu *et al.*, 2013), MalR from *Corynebacterium glutamicum* ATCC 13032 (Krause *et al.*, 2012), SCO3205 from *S. coelicolor* A3(2) (Stevenson *et al.*, 2013; PDB, 3ZPL), BadR from *R. palustris* CGA009 (Egland and Harwood, 1999), AdcR from *Streptococcus pneumoniae* D39 (Ogunniyi *et al.*,

2009; PDB, 3TGN), HpaR from *E. coli* W (Prieto *et al.*, 1996), CinR from *Butyrivibrio fibrisolvens* E14 (Dalrymple and Swadling, 1997), MexR from *Pseudomonas aeruginosa* PAO1 (Evans *et al.*, 2001; PDB, 1LNW), and ChlR from *Synechococcus* sp. PCC 7002 (Ludwig *et al.*, 2014). Comparison was performed using Clustal Omega (http://www.ebi.ac.uk/Tools/msa/clustalo/). The clade that CouR from *R. jostii* RHA1 belongs to is coloured yellow.

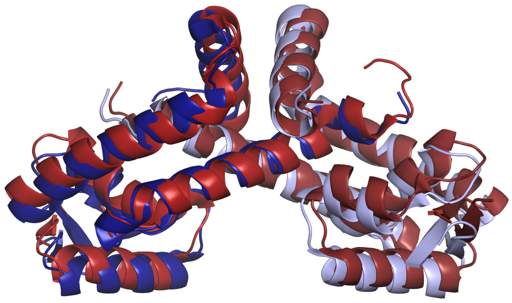

Fig. S4. Secondary structure superposition of the two apo-CouR dimers of the asymmetric unit. Chains A and C, and Chains B and D are shown in red and blue, respectively. The αC RMSD value was 0.70 Å over 115 matching α-carbon atoms.

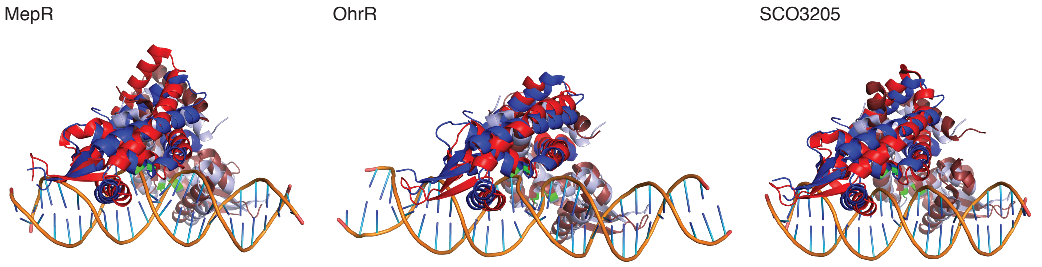

Fig. S5. Structural alignment of CouR (deep blue), and *S. aureus* MepR, *B. suttilis* OhrR, or *S. corlicolor* A3(2) SCO3205 (red) bound to DNA. Arg36 and Arg38 are shown in green. The αC RMSD values were 3.42 Å (255 matching α-carbon atoms), 3.48 Å (254 matching α-carbon atoms) or 3.26 Å (249 matching α-carbon atoms) when superimposed on the MepR-DNA, OhrR-DNA or SCO3205-DNA structure, respectively.

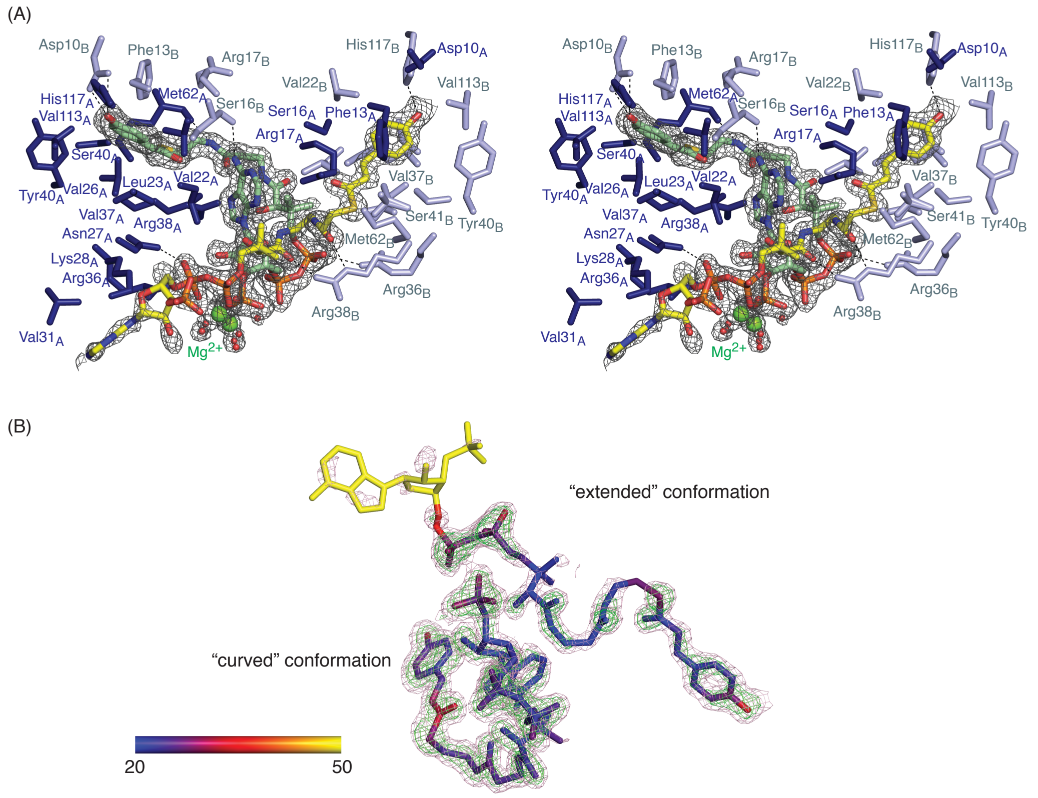

Fig. S6. Structure of the ligand-bound form of CouR. (A) Stereo image of an electron density map for *p*-coumaroyl-CoA ligands bound to CouR. Map shown is a simple F_o-F_c (omit) map at 3.0 σ. Sticks represent ligand atoms and CouR residues comprising active site cleft, coloured differently according to the two chains in the protein:ligand complex dimer. Green spheres indicate Mg^{2+} ions bound to phosphates of the ligands. (B) The structure of *p*-coumaroyl-CoA coloured by its B-factor. Map shown is a simple F_o-F_c (omit) map at 3.0 σ (light pink) or 5.0 σ (green).

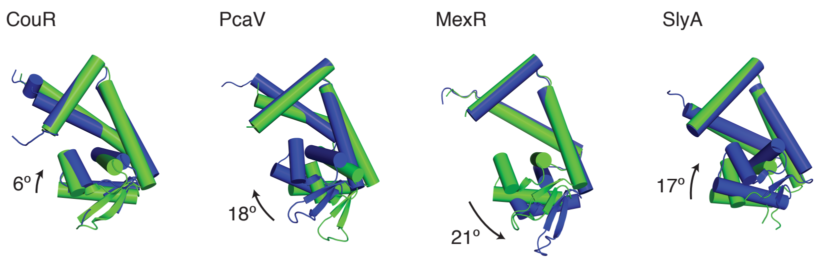

Fig. S7. Superposition of the dimerisation core of one monomer of apo- (blue) and ligand-bound (green) CouR, PcaV (4G9Y and 4FHT), MexR (1LNW and 3ECH), and SlyA (3QPT and 3DEU). Their DNA binding domains rotate up to 6º, 18º, 21º or 17º, respectively.

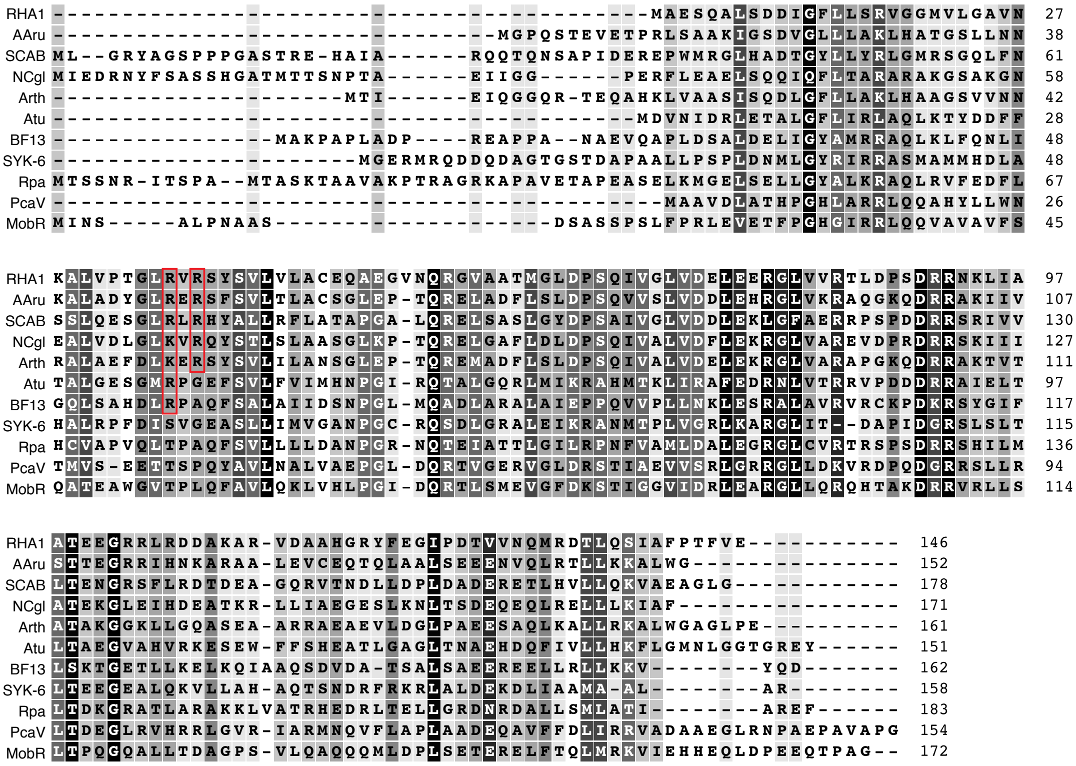

Fig. S8. Amino acid sequence alignment of CouR and its homologues: RHA1, CouR from *R. jostii* RHA1; AAru, AAur0960 from *Arthrobacter aurescence* TC1; SCAB, SCAB15631 from *Streptomyces scabiei* 87.22; NCgl, NCgl0280 from *Corynebacterium glutamicum* ATCC13032; Arth, Arth3825 from *Arthrobacter* sp. FB24; Atu, Atu1422 from *Agrobacterium fabrum* C58; BF13, FerR from *P. fluorescens* BF13; SYK-6, FerC from *Sphingobium* sp. SYK-6; Rpa, CouR from *R. palustris* CGA009; PcaV from *S. coelicolor* A3(2); and MobR from *C. testosteroni* KH122-3s. Sequences were aligned using Clustal Omega (http://www.ebi.ac.uk/Tools/msa/clustalo/). The Arg36 and Arg38 of $CouR_{RHA1}$ and their equivalent residues are indicated by red boxes.

Table S1. Oligonucleotides used in this study.

Name	Sequence (5' to 3')	Purpose
*couR*_FF	CCGGAATTCTCACTTCGCCATCAGCTCC	*couR* deletion
*couR*_FR	GGTGGGAAAGGCGATGGGTACGAGGGCTTTGTTCA	*couR* deletion
*couR*_RF	AAAGCCCTCGTACCCATCGCCTTTCCCACCTTCGT	*couR* deletion
*couR*_RR	CCCAAGCTTAGGTACTCCACCGAGATCTG	*couR* deletion
*couR*_c_F	TGCTCTAGACACCAGGTCCTTGGCGTATG	Complementation
*couR*_c_R	CCGGAATTCGAGCTGAGCAGCTGGAAGTC	Complementation
*couR*_F	GGAATTCCATATGGCAGAGTCGCAGGCGTT	CouR purification
*couR*_R	CGCGGATCCCTACTCCACGAAGGTGGGAA	CouR purification
*couR*36A_S	CCCACGGGTCTGGCGGTGCGCTCCTA	Generation of R36A
*couR*36A_A	TAGGAGCGCACCGCCAGACCCGTGGG	Generation of R36A
*couR*38A_S	GGTCTGCGGGTGGCCTCCTACTCGGT	Generation of R38A
*couR*38A_A	ACCGAGTAGGAGGCCACCCGCAGACC	Generation of R38A
*couR*36A38A_S	ACCCACGGGTCTGGCGGTGGCCTCCTACTCGGTC	Generation of R36A/R38A
*couR*36A38A_A	GACCGAGTAGGAGGCCACCGCCAGACCCGTGGGT	Generation of R36A/R38A
*couR*p_*couN*p_S	CATTGATGATTGAGAATGTCAATGATTAAGG	EMSA
*couR*p_*couN*p_A	CCTTAATCATTGACATTCTCAATCATCAATG	EMSA
*couR*p_*couN*p_m_S	CATTGATGTAACTGAATGAGTTAGATTAAGG	EMSA
*couR*p_*couN*p_m_A	CCTTAATCTAACTCATTCAGTTACATCAATG	EMSA
*couT*p_S	CATTAATCATTGAACTTGTCAATGGAAGAGA	EMSA
*couT*p_A	TCTCTTCCATTGACAAGTTCAATGATTAATG	EMSA
*couH*p_S	TATCAATCATTGATAATGTCAATGATTGACT	EMSA
*couH*p_A	AGTCAATCATTGACATTATCAATGATTGATA	EMSA
*sigA*_q-F	CCAAGGAAATGGACATCACG	qRT-PCR
*sigA*_q-R	ATCGTCTGGTCGAGGGAGAT	qRT-PCR
*couR*_q_F	CAGAGTCGCAGGCGTTATC	qRT-PCR
*couR*_q_R	GTACGAGGGCTTTGTTCACC	qRT-PCR

Table S2. Strains and plasmids used in this study.

	Description	Source or reference
Escherichia coli		
DH5α	F^-, Φ80d*lacZ*ΔM15, Δ(*lacZYA-argF*)U169, *deoR*, *recA*1, *endA*1, *hsdR*17(r_K^-, m_K^+), *phoA*, *supE*44, λ^-, *thi*-1, *gyrA*96, *relA*1	Bethesda Research
BL21(DE3)	*hsdS gal*(λ*cIts857 indI Sam7 nin5 lac*UV5-T7 *gene1*)	(Green and Sambrook, 2012)
Rhodococcus jostii		
RHA1	Wild-type	(Seto *et al*., 1995)
Δ*couR*	*couR* mutant	This study
Δ*couR*::pSET*couR*	*R. jostii* Δ*couR* bearing pSET*couR*	This study
Δ*couR*::pSET152	*R. jostii* Δ*couR* bearing pSET152	This study
Δ*couR*::pSET*couR*36A38A	*R. jostii* Δ*couR* bearing pSET*couR*36A38A	This study
**Plasmid**		
pK18Δ*couR*	*couR* deletion plasmid; *sacB*, *oriT*, *aphII*	This study
pSET152	Actinomycete integrating vector; *lacZ*α, rep^{pUC}, *aac(3)IV*, $int^{\phi C31}$, *oriT*	(Bierman *et al*., 1992)
pSET*couR*	pSET152 harboring *couR* gene	This study
pSET*couR*36A38A	pSET152 harboring *couR*_R36AR38A gene	This study
pCo*couR*	pColdI harboring the coding sequence of *couR*	This study
pCo*couR*36A	pColdI harboring the coding sequence of *couR*_R36A	This study
pCo*couR*38A	pColdI harboring the coding sequence of *couR*_R38A	This study
pCo*couR*36AR38A	pColdI harboring the coding sequence of *couR*_R36AR38A	This study

References

Abomoelak,B., Hoye,E.A., Chi,J., Marcus,S.A., Laval,F., Bannantine,J.P., Ward,S.K., Daffe,M., Liu,H.D. and Talaat,A.M. (2009) mosR, a novel transcriptional regulator of hypoxia and virulence in *Mycobacterium tuberculosis*. *J. Bacteriol.*, **191**, 5941-5952.

Bierman,M., Logan,R., O'Brien,K., Seno,E.T., Rao,R.N. and Schoner,B.E. (1992) Plasmid cloning vectors for the conjugal transfer of DNA from *Escherichia coli* to *Streptomyces* spp. *Gene*, **116**, 43-49.

Calisti,C., Ficca,A.G., Barghini,P. and Ruzzi,M. (2008) Regulation of ferulic catabolic genes in *Pseudomonas fluorescens* BF13: involvement of a MarR family regulator. *Appl. Microbiol. Biotechnol.* **80**, 475– 483.

Davis,J.R., Brown,B.L., Page,R. and Sello,J.K. (2013) Study of PcaV from *Streptomyces coelicolor* yields new insights into ligand-responsive MarR family transcription factors. *Nucleic Acids Res*., **41**, 3888-3900.

Dalrymple,B.P. and Swadling,Y. (1997) Expression of a *Butyrivibrio fibrisolvens* E14 gene (*cinB*) encoding an enzyme with cinnamoyl ester hydrolase activity is negatively regulated by the product of an adjacent gene (*cinR*). *Microbiology*, **143**, 1203-1210.

Dolan,K.T., Duguid,E.M. and He,C. (2011) Crystal structures of SlyA protein, a master virulence regulator of *Salmonella*, in free and DNA-bound states. *J. Biol. Chem.*, **286**, 22178-22185.

Egland,P.G. and Harwood,C.S. (1999) BadR, a new MarR family member, regulates anaerobic benzoate degradation by *Rhodopseudomonas palustris* in concert with AadR, an Fnr family member. *J. Bacteriol.*, **181**, 2102-2109.

Evans,K., Adewoye,L. and Poole,K. (2001) MexR repressor of the *mexAB-oprM* multidrug efflux operon of *Pseudomonas aeruginosa*: Identification of MexR binding sites in the *mexA-mexR* intergenic region. *J. Bacteriol.*, **183**, 807–812.

Fuangthong, M., Atichartpongkul,S., Mongkolsuk,S. and Helmann,J.D. (2001) OhrR is a repressor of *ohrA*, a key organic hydroperoxide resistance determinant in *Bacillus subtilis*. *J. Bacteriol.*, **183**, 4134-4141.

Green,M.R., and Sambrook,J. (2012) Molecular Cloning: A Laboratory Manual (Fourth edition). Cold Spring Harbor Laboratory Press, Cold Spring Harbor, NY.

Hirakawa,H., Schaefer,A.L., Greenberg,E.P. and Harwood,C.S. (2012) Anaerobic *p*-coumarate degradation by *Rhodopseudomonas palustris* and identification of CouR, a MarR repressor protein that binds *p*-coumaroyl-CoA. *J. Bacteriol.* **194**, 1960–1967.

Hiromoto,T., Matsue,H., Yoshida,M., Tanaka,T., Higashibata,H., Hosokawa,K., Yamaguchi,H. and Fujiwara,S. (2006) Characterization of MobR, the 3-hydroxybenzoate-responsive transcriptional regulator for the 3-hydroxybenzoate hydroxylase gene of *Comamonas testosteroni* KH122-3s. *J. Mol. Biol.*, **364**, 863-877.

Huang,H. and Grove,A. (2013) The transcriptional regulator TamR from *Streptomyces coelicolor* controls a key step in central metabolism during oxidative stress. *Mol. Microbiol.* **87**, 1151–1166.

Huang,H., Mackel,B.J. and Grove,A. (2013) *Streptomyces coelicolor* encodes a urate-responsive transcriptional regulator with homology to PecS from plant pathogens. *J. Bacteriol.*, **195**, 4954-4965.

Kasai,D., Kamimura,N., Tani,K., Umeda,S., Abe,T., Fukuda,M. and Masai,E. (2012) Characterization of FerC, a MarR-type transcriptional regulator, involved in transcriptional regulation of the ferulate catabolic operon in *Sphingobium* sp. strain SYK-6. *FEMS Microbiol. Lett.*, **332**, 68-75.

Krause,J.P., Polen,T., Youn,J.W., Emer,D., Eikmanns,B.J. and Wendisch,V.F. (2012) Regulation of the malic enzyme gene *malE* by the transcriptional regulator MalR in *Corynebacterium glutamicum*. *J. Biotechnol.*, **159**, 204-215.

Kumaraswami,M., Schuman,J.T., Seo,S.M., Kaatz,G.W. and Brennan,R.G. (2009) Structural and biochemical characterization of MepR, a multidrug binding transcription regulator of the *Staphylococcus aureus* multidrug efflux pump MepA. *Nucleic Acids Res.*, **37**, 1211-1224.

Lomovskaya,O., Lewis,K. and Matin,A. (1995) EmrR is a negative regulator of the *Escherichia coli* multidrug resistance pump EmrAB. *J. Bacteriol.*, **177**, 2328-2334.

Ludwig,M., Pandelia,M.E., Chew,C.Y., Zhang,B., Golbeck,J.H., Krebs,C. and Bryant,D.A. (2014) ChlR protein of *Synechococcus* sp. PCC 7002 is a transcription activator that uses an oxygen-sensitive [4Fe-4S] cluster to control genes involved in pigment biosynthesis. *J. Biol. Chem.*, **289**, 16624-16639.

Martin,R.G., Jair,K.W., Wolf,R.E.,Jr. and Rosner,J.L. (1996) Autoactivation of the marRAB multiple antibiotic resistance operon by the MarA transcriptional activator in *Escherichia coli*. *J. Bacteriol.*, **178**, 2216-2223.

Newberry,K.J., Fuangthong,M., Panmanee,W., Mongkolsuk,S. and Brennan,R.G. (2007). Structural mechanism of organic hydroperoxide induction of the transcription regulator OhrR. *Mol. Cell*, **28**, 652–664.

Ogunniyi,A.D., Grabowicz,M., Mahdi,L.K., Cook,J., Gordon,D.L., Sadlon,T.A. and Paton,J.C. (2009) Pneumococcal histidine triad proteins are regulated by the Zn^{2+}-dependent repressor AdcR and inhibit complement deposition through the recruitment of complement factor H. *FASEB J.*, **23**, 731-738.

Parke,D. and Ornston,L.N. (2003) Hydroxycinnamate (*hca*) catabolic genes from *Acinetobacter* sp. strain ADP1 are repressed by HcaR and are induced by hydroxycinnamoyl-coenzyme A thioesters. *Appl. Environ. Microbiol.*, **69**, 5398-5409.

Prieto,M.A., Diaz,E. and Garcia,J.L. (1996) Molecular characterization of the 4-hydroxyphenylacetate catabolic pathway of *Escherichia coli* W: engineering a mobile aromatic degradative cluster. *J. Bacteriol.*, **178**, 111-120.

Radhakrishnan,A., Kumar,N., Wright,C.C., Chou,T.H., Tringides,M.L., Bolla,J.R., Lei,H.T., Rajashankar,K.R., Su,C.C., Purdy,G.E. *et al.* (2014) Crystal structure of the transcriptional regulator Rv0678 of *Mycobacterium tuberculosis*. *J. Biol. Chem.*, **289**, 16526-16540.

Providenti,M.A. and Wyndham,R.C. (2001) Identification and functional characterization of CbaR, a MarR-like modulator of the *cbaABC*-encoded chlorobenzoate catabolism pathway. *Appl. Environ. Microbiol.*, **67**, 3530-3541.

Rouanet,C., Reverchon,S., Rodionov,D.A. and Nasser,W. (2004) Definition of a consensus DNA-binding site for PecS, a global regulator of virulence gene expression in *Erwinia chrysanthemi* and identification of new members of the PecS regulon. *J. Biol. Chem.*, **279**, 30158-30167.

Rüberg,S., Pühler,A. and Becker,A. (1999) Biosynthesis of the exopolysaccharide galactoglucan in *Sinorhizobium meliloti* is subject to a complex control by the phosphate-dependent regulator PhoB and the proteins ExpG and MucR. *Microbiology*, **145**, 603-611.

Seto,M., Kimbara,K., Shimura,M., Hatta,T., Fukuda,M. and Yano,K. (1995) A Novel Transformation of Polychlorinated Biphenyls by *Rhodococcus* sp. Strain RHA1. *Appl. Environ. Microbiol.*, **61**, 3353-3358.

Stevenson,C.E., Assaad,A., Chandra,G., Le,T.B., Greive,S.J., Bibb,M.J. and Lawson,D.M. (2013) Investigation of DNA sequence recognition by a streptomycete MarR family transcriptional regulator through surface plasmon resonance and X-ray crystallography. *Nucleic Acids Res.*, **41**, 7009-7022.

Wilkinson,S.P. and Grove,A. (2004) HucR, a novel uric acid-responsive member of the MarR family of transcriptional regulators from *Deinococcus radiodurans*. *J. Biol. Chem.*, **279**, 51442-51450.

Zhu,D., Wang,Y., Zhang,M., Ikeda,H., Deng,Z. and Cane,D.E. (2013) Product-mediated regulation of pentalenolactone biosynthesis in *Streptomyces* species by the MarR/SlyA family activators PenR and PntR. *J. Bacteriol.*, **195**, 1255-1266.
